# The Neuroinfectious diseases profile in a specialist neurology centre in South Africa

**DOI:** 10.1016/j.ensci.2025.100581

**Published:** 2025-08-06

**Authors:** Katryn Oosthuizen, Suzaan Marais

**Affiliations:** a Division of Neurology, Department of Medicine, University of Cape Town, Western Cape, South Africa; b Neurology Research Group, Neuroscience Institute, University of Cape Town, Western Cape, South Africa

**Keywords:** Neuroinfectious disease, Neurologic infection, HIV, Tuberculosis, Tuberculous meningitis, Syphilis, Neurosyphilis, COVID-19

## Abstract

**Objective:**

This study aimed to assess the burden of neuroinfectious diseases and describe the causes and presentation of neurological infections to a tertiary level hospital neurology service in South Africa.

**Methods:**

A retrospective electronic search of medical discharge records was conducted for adult patients admitted to the neurology ward over a two-year period, and patients with neuroinfectious diseases were identified. Diagnostic criteria were applied to classify patients according to the certainty of their neuroinfectious disease diagnosis.

**Results:**

Neuroinfectious diseases accounted for 15 % of the 802 admissions to the neurology ward. The most common infectious aetiologies were tuberculosis (27 %), syphilis (21 %), neurological diseases related to human immunodeficiency virus (HIV) itself (19 %), and HIV-associated opportunistic infections (10 %). Diagnostic challenges were observed, with only 17 % of cases having a definite diagnosis. The majority of patients with neuroinfectious diseases were young, with a median age of 38 years (IQR: 32–46), and 56 % were HIV-positive. Morbidity was high, with prolonged hospitalisation (>2 weeks) and limited full recovery at discharge in 56 % and 98 % of patients, respectively.

**Conclusions:**

This study provides important insights into the burden and characteristics of neuroinfectious diseases encountered at an inpatient South African neurology service. The findings highlight the need for increased investment in primary care prevention and treatment of HIV, TB and syphilis − before patients require hospital admission or develop neurological pathology.

## Highlights

• Neuroinfectious diseases accounted for 15 % of all admissions.
• Most patients with a neuroinfectious disease were HIV-positive.
• HIV, TB, and other opportunistic infections caused the majority of neurological infections.
• Only 17 % of cases could be classified as having a definite diagnosis.
• Neuroinfectious diseases were associated with prolonged hospitalisation and high morbidity.

## 1 Introduction

There are more than 100 pathogens including viruses, bacteria, parasites and fungi that can damage the human central or peripheral nervous system, either directly or through post-infectious inflammatory responses [1]. The three most important global infectious diseases –malaria, tuberculosis (TB), and the human immunodeficiency virus (HIV)– frequently cause neurological complications [2,3]. Furthermore, emerging diseases such as severe acute respiratory syndrome coronavirus 2 (SARS-CoV-2), increasing proportions of drug resistant organisms, and an increasing number of immunosuppressed populations, all contribute to the global burden of neurological infectious diseases [[2], [3], [4], [5], [6]].

Infections may not only result in severe acute neurological deficits, but may also have devastating neurological sequelae for those who survive the initial illness [1,7]. Neurological infections are often associated with high mortality rates and can cause severe disability [8]. This consequently leads to a significant social and economic burden on individuals, families, and healthcare systems. Furthermore, the burden of neurological infectious diseases is unevenly distributed, with low- and middle-income countries (LMICs) most severely affected [5,9]. Reflecting, at least in part, the limited availability of specialist neurological services in the developing world, minimal research has been conducted on the burden of neurological infections in specialist neurological centres in LMIC, despite the higher burden of infectious diseases in these regions [5,9].

This study aimed to assess the burden of neuroinfectious diseases, and to describe the causes and presentation of neurological infections, in a tertiary hospital neurology service in South Africa.

## 2 Methods

### 2.1 Patient identification

We performed a retrospective electronic search of medical discharge records of all adult patients (age: ≥ 18 years) admitted to the neurology ward at Groote Schuur Hospital (GSH) in Cape Town, South Africa, over a two-year period (from 1 February 2020 through January 2022).

Patients with a primary or secondary ICD-10 diagnostic code indicating a neuroinfectious disease or another systemic infectious disease that has well-recognised risks of neurological sequalae, such as HIV, were identified. The medical records of these patients, in conjunction with laboratory and radiological data, were then systematically reviewed to identify those who met criteria for various neuroinfectious diseases. We included patients in the neuroinfectious disease (NID) group if they met predefined diagnostic criteria (Appendix A). Diagnostic criteria were applied to neurological infections in general, as well as to specific infections including neurosyphilis, neurological TB, progressive multifocal leukoencephalopathy (PML), neurological disease associated with coronavirus disease 2019 (COVID-19), neuroschistosomiasis, neurocysticercosis, HIV-associated neurocognitive disorder (HAND), and cerebral toxoplasmosis. Criteria were used to subclassify patients based on the diagnostic certainty of their NID as definite, probable or possible.

We included HIV-positive patients in the NID group if HIV was deemed causal or contributing to their neurological condition, and excluded HIV-positive patients if their neurological condition was not attributed to HIV. Patients were also excluded from the NID group if any of the following criteria were met: 1) An alternative cause was identified to explain the clinical presentation; 2) Clinical improvement occurred without appropriate therapy (where relevant); 3) Clinical records were incomplete.

As per our hospital laboratory protocol, the initial serological screening test for syphilis was a *Treponema pallidum* serum antibody (TpAb) test. If the TpAb test was positive or equivocal, the nontreponemal serum rapid plasma reagin (RPR) test was performed. Patients were considered to have active syphilis if they had a positive serum RPR test, in the context of a positive or equivocal serum TpAb test, or were diagnosed with neurosyphilis. A definite diagnosis of neurosyphilis was made in patients with a reactive cerebrospinal fluid (CSF) VDRL (venereal disease research laboratory) test; diagnostic criteria for possible and probable neurosyphilis are detailed in Appendix A.

### 2.2 Statistical analysis

Statistical analyses were performed using GraphPad Prism® software. Continuous data were presented as medians with interquartile ranges and compared using the Mann-Whitney *U* test. Categorical variables were presented as frequencies with percentages and compared using Fisher's exact test. A *p*-value of <0.05 was considered statistically significant.

## 3 Results

**Fig. 1:**
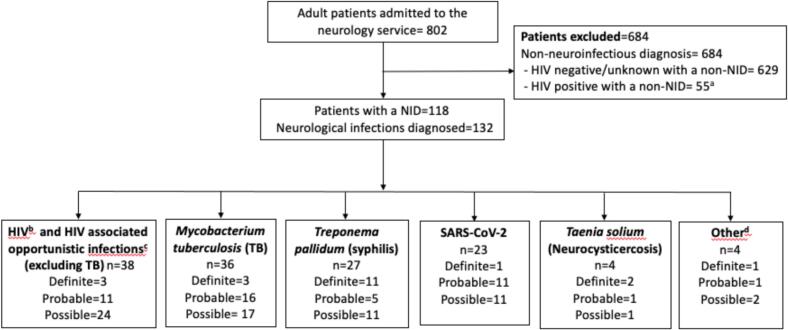
Flow diagram of adult patients admitted to the neurology service over a two-year period. ^**a**^ Patients who were human immunodeficiency virus (HIV) positive but with no noted neurological manifestations of HIV. ^**b**^ HIV associated neurological disease: HIV-associated neurocognitive disorder (HAND), HIV associated polyneuropathy, HIV vacuolar myelopathy, CD8 encephalitis, polymyositis, central nervous system (CNS) vasculopathy/ vasculitis, ischaemic stroke with no other cause. ^**c**^**Opportunistic** infections: John Cunningham virus (JCV), *Toxoplasma gondii,* Cytomegalovirus (CMV). ^**d**^ Other infections: *Schistosoma haematobium,* Varicella zoster virus, *Streptococcus pneumoniae, Listeria monocytogenes.* Diagnostic criteria used to classify a NID diagnosis as definite, probable or possible are defined in Appendix A. Abbreviations:  NID: Neuroinfectious disease SARS-CoV-2:  Severe acute respiratory syndrome coronavirus 2

During the two-year study period, 802 adults were admitted to the neurology ward at GSH. Fig. 1 shows the reasons for the exclusion and inclusion of the study. Fifteen percent of the patients (*n* = 118) had a NID and a total of 132 cases of NID were diagnosed; 14 patients fulfilling criteria for the diagnosis of two concurrent neurological infections. Of the 132 NID cases diagnosed, 22 (17 %) were classified as a definite diagnosis, 47 (36 %) as a probable, and 63 (48 %) as a possible NID diagnosis.

### 3.1 Baseline characteristics of patients with; and without; a neuroinfectious disease

Characteristics of patients diagnosed with an NID and those diagnosed with a non-neuroinfectious disease (non-NID) are summarised in Table 1. An equal number of male and female patients (59 each) were admitted with a NID. Patients diagnosed with NID were significantly younger (median age: 38 years [IQR 32–46]), than patients in the non-NID group (48-years [37–60]; *p* < 0.001).

**Table 1:** Clinical characteristics of the 802 patients admitted to the neurology service during the study period.

	**Neuroinfectious disease;** **n** **=** **118**		**Non-neuroinfectious disease;** ***n*** **=** **684**		***P* value**
	Number (%)	Number of patients	Number (%)	Number of patients	
Female sex	59 (50)	118	362 (53)	684	0.618
Age (years) Median (IQR)	38 (32–46)	118	48 (37–60)	684	<0.001
Admission duration (days) Median (IQR)	16 (9–28)	118	10 (6–18)	684	<0.001
In-hospital mortality	6 (5)	118	27 (4)	684	0.614
HIV positive Female	66 (56) 45 (68)	118 66	55 (9) 34 (62)	612 55	<0.001 0.566
CD4 count <200 cells/μL)	36 (55)	66	10 (19)	53	<0.001
On ART	35 (53)	66	37 (67)	55	0.138
Syphilis exposure^a^	36 (31)	118	47 (8)	601	<0.001
Active syphilis^b^	28 (24)	118	18 (3)	601	<0.001
COVID-19 positive^c^	23 (27)	86	18 (4)	457	<0.001

Continuous variables were compared using the Mann-Whitney U test; categorical variables were compared using Fisher's two-sided exact test; a *p*-value of <0.05 was considered statistically significant.

Abbreviations: HIV: Human Immunodeficiency virus, ART: Anti-retroviral therapy, COVID-19: Corona Virus Disease of 2019.

a Patients classified as syphilis exposed if they had a positive serum/ CSF treponemal test.

b Active syphilis diagnosed if patients had a positive serum treponemal test and a positive or equivocal serum non-treponemal test, or if they were diagnosed with neurosyphilis.

c Number of patients (543) for whom COVID-19 test results were available.

### 3.2 Screening for infectious diseases

Ninety-one percent (*n* = 730) of all admitted patients were tested for HIV, including all patients with a NID and 612 (90 %) of those with a non-NID (Table 1). The overall HIV prevalence of patients who were tested was 17 % (*n* = 121), including 56 % (*n* = 66) of patients in the NID and 9 % (*n* = 55) of patients tested in the non- NID group. The HIV prevalence was higher among women than among men. Among women with a NID the HIV prevalence was 76 % compared to 36 % in men, whilst in the non-NID group the prevalence was 11 % in women and 7 % in men. CD4 counts from HIV-positive patients were lower in the NID-group (median: 167cells/μL [IQR 86–344]) compared to those of the non-NID group (375cells/mm3 [237–690]; p < 0.001). Forty-seven percent (*n* = 31) of HIV-positive patients in the NID group were not receiving antiretroviral therapy (ART) prior to presentation; of these 17 patients were newly diagnosed with HIV, 10 having interrupted ART and four were previously diagnosed but were ART-naïve. Of the 31 NID patients who were receiving ART and had their HIV viral loads tested, 22 (71 %) had a suppressed serum viral load (<50 copies/ml).

All patients admitted with an NID were tested for syphilis, with 31 % (*n* = 36) testing positive on a serum or CSF treponemal test (Table 1). Of the 802 total study patients (NID and non-NID), 719 (90 %) were tested for syphilis. Among these 719 patients, the overall prevalence of exposure to syphilis was 12 % (*n* = 83). Syphilis exposure was higher in tested males (51/345; 15 %) than in tested females (32/374; 9 %; *p* = 0.021). One hundred-and-nineteen (98 %) of the 121 HIV-positive patients, and 587 (96 %) of the 609 confirmed HIV-negative patients, were tested for syphilis. Syphilis exposure among HIV-positive patients was greater (25/119; 21 %) compared to HIV-negative patients (57/609; 9 %; *p* < 0.001). Among the patients tested for syphilis, 27 were eventually diagnosed with neurosyphilis. Of the remaining patients screened for syphilis, 3 % (19/692) had active syphilis other than neurosyphilis; their diagnosis of syphilis was incidental, asymptomatic, and unrelated to their presenting disease.

As patients presented during or shortly after the COVID-19 pandemic, the majority of patients were tested for SARS-CoV-2 infection (*n* = 543, 68 %). Forty-one (8 %) tested positive by the SARS-CoV-2 polymerase chain reaction (PCR) performed on nasopharyngeal samples (Table 1).

### 3.3 Classification of neuroinfectious diseases

**Fig. 2:**
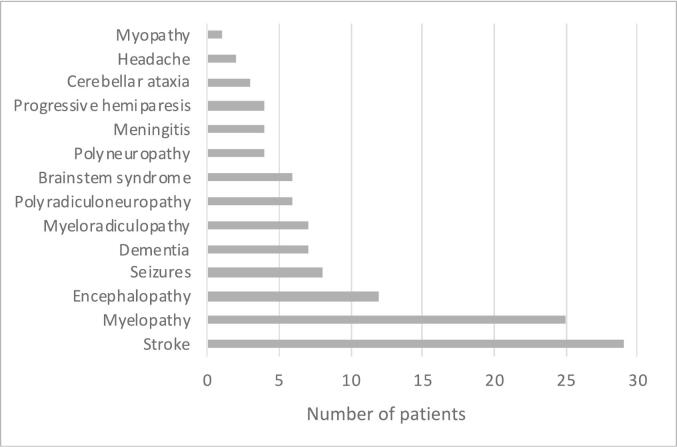
Clinical presentation features in patients with a neuroinfectious disease (*n* = 118).

The presenting clinical syndromes of the 118 patients admitted with an NID are illustrated in Fig. 2.

The most common presentations for the patients were strokes (*n* = 29; 25 %), myelopathy (*n* = 25; 21 %), encephalopathy (*n* = 12; 10 %) and seizures (n = 8; 7 %).

Infectious aetiologies are summarised in Table 2. Together *Mycobacterium tuberculosis* (n = 36; 27 %), *treponema pallidum* (*n* = 27; 21 %) and neurological disease related to HIV itself (*n* = 25; 19 %) caused more than two thirds of the 132 NID cases identified.

**Table 2:** Aetiologies of the 132 diagnosed neuroinfectious diseases.

*Mycobacterium tuberculosis*	n = 36 (27 %)
Meningitis	10
Non-bony myeloradiculitis	16
Bony TB spine	2
Tuberculomas	5
Ischaemic stroke	2
TB associated polyneuropathy	1
HIV	n = 25 (19 %)
HAND	6
CNS vasculopathy/ vasculitis	6
HIV vacuolar myelopathy	3
Strokes attributed to HIV thrombophilia	3
HIV associated polyneuropathy	3
CD8 encephalitis	2
DILS associated radiculoneuropathy	1
Polymyositis	1
HIV associated opportunistic infections^a^	*n* = 13 (10 %)
John Cunningham virus	9
*Toxoplasma gondii*	2
Cytomegalovirus	2
*Treponema pallidum*	n = 27 (21 %)
Meningovascular	12
Myelopathy	12
Neuropsychiatric	1
Incidental^b^	2
SARS-CoV-2	*n* = 23 (17 %)
Cerebral infarcts	9
Meningoencephalitis	6
Spinal cord infarcts	2
Dural venous sinus thrombosis	2
Post-COVID Guillain-Barre syndrome	4
Axonal polyneuropathy	1
*Taenia solium*	n = 4 (3 %)
Other^c^	*n* = 4 (3 %)

Abbreviations: HIV: Human Immunodeficiency virus, HAND: HIV associated neurocognitive disorder, DILS: Diffuse infiltrative lymphocytosis syndrome, SARS-COV-2: Severe acute respiratory syndrome coronavirus 2, COVID-19: Corona Virus Disease of 2019.

a Opportunistic infections excluding tuberculosis (TB).

b Neurosyphilis diagnoses deemed incidental as patients had symptomatic polyneuropathies due to HIV and GBS (Guillain-Barre syndrome);

c Other infections: *Listeria monocytogenes* n = 1, *Schistosoma haematobium* n = 1, *Streptococcus pneumoniae* (bacterial meningitis) n = 1, Varicella zoster *n* = 1.

#### 3.3.1 Neurological TB

Of the 36 cases diagnosed as neurological TB, a definite diagnosis was only possible in three patients (Fig. 1): Two patients had positive CSF GeneXpert MTB/RIF Ultra assays and one patient had a biopsy-proven spinal tuberculoma. A probable and possible diagnosis of neurological TB was made in 16 and 17 patients, respectively. Most of the patients with neurological TB were HIV-positive (*n* = 25; 69 %), with a median CD4 count of 137 cells/μL (IQR 105–289). Concurrent pulmonary TB was confirmed by sputum testing in six (17 %) patients, and suspected in four additional patients according to radiographic characteristics on chest computed tomography (CT). Abdominal ultrasound imaging demonstrating splenic microabscesses suggestive of extrapulmonary TB was evident in six patients, two of whom also had a positive urine lipoarabinomannan (LAM) test. Non-bony TB myeloradiculitis was diagnosed in 16 patients. Two patients had bony TB spine disease (Pott's disease). TB meningitis occurred in 10 cases (28 %) and intracranial tuberculomas in five (14 %).

#### 3.3.2 HIV and opportunistic infections

Thirty-seven patients had a NID related to HIV itself or to opportunistic infections other than TB and of these, ten (27 %) were diagnosed with HIV at presentation. The median CD4 count (known in all patients) was 146 cells/μL (IQR 62–356). Of 25 cases that were related to HIV itself, HAND and HIV associated CNS vasculitis/ vasculopathy were the most frequently encountered conditions, each affecting six patients. Furthermore, 13 HIV-positive patients in the NID group presented with opportunistic infections other than TB, including John Cunningham virus (JCV) causing PML (*n* = 9), *Toxoplasma gondii* related encephalitis (n = 2), Cytomegalovirus (CMV) related myeloradiculopathy, and CMV encephalitis respectively (n = 2).

#### 3.3.3 Neurosyphilis

The diagnostic criteria for possible, probable and definite neurosyphilis are detailed in Appendix A. Of the patients diagnosed with neurosyphilis, 41 % (11/27) had a definite diagnosis of neurosyphilis, as evidenced by a neurological manifestation consistent with neurosyphilis and a reactive CSF VDRL test. The remaining patients had probable (*n* = 5) or possible (*n* = 11) neurosyphilis. Twenty (74 %) of the neurosyphilis patients were male. All 27 patients treated for neurosyphilis were tested for HIV, of whom nine (33 %) were HIV-positive. Meningovascular syphilis and syphilitic myelopathy were equally common, affecting 12 patients and together contributing to 89 % (*n* = 24) of cases. The most common manifestation of meningovascular syphilis was ischaemic strokes, diagnosed in 7 patients, with the others having meningitis (n = 5). An incidental diagnosis of probable and possible neurosyphilis was made in two patients with polyradiculoneuropathy, whose primary neurological diagnoses were Guillain-Barre syndrome (GBS) and diffuse infiltrative lymphocytosis syndrome (DILS), respectively.

#### 3.3.4 SARS-CoV-2

Of 23 patients diagnosed with a NID caused by SARS-CoV-2, a definite diagnosis was only possible in a single patient with encephalitis presenting as a clinical cerebellar syndrome, in whom the diagnosis was based on a positive CSF SARS-CoV-2 PCR test. The details of this case were previously published [10]. An equal number of patients (11 each) met the possible and probable diagnostic criteria for neurological disease associated with SARS-CoV-2. The manifestation most frequently encountered was thrombophilia, resulting in ischaemic strokes (9/23; 39 %) and meningoencephalitis (6/23; 26 %). Diagnostic criteria for COVID-19 associated neurological syndromes are detailed in Appendix A.

#### 3.3.5 Other infections

Uncommon infections diagnoses included: Neurocysticercosis diagnosed in four patients, and bacterial meningitis due to *Streptococcus pneumoniae*, encephalitis due to *Listeria monocytogenes*, myelitis due to *Schistosoma haematobium,* and myelitis due to varicella zoster virus (VZV), diagnosed in one patient each.

#### 3.3.6 Dual infections

Fourteen patients were treated for two concurrent neurological infections, of which 12 patients were HIV-positive. Six patients were treated for neurosyphilis and TB, with four of these patients presenting with myelopathies and the remaining two patients presenting with rhombencephalitis, and meningitis, respectively. Two patients were diagnosed with neurosyphilis and a primary HIV- associated neurological disease; one patient had HAND, and the other had a polyneuropathy. One patient was diagnosed with both HAND and a DILS radiculoneuropathy. Two HIV-positive patients with ring-enhancing lesions on neuroimaging were treated simultaneously for both TB and toxoplasmosis. Two patients with myelitis were treated for TB, in addition to receiving antiviral treatment for CMV and VZV, respectively. One patient with encephalitis was treated for CMV and JCV infection as both CMV and JCV CSF PCR tests were positive. A young patient with HIV vasculopathy had a stroke in the context of COVID-associated thrombophilia.

### 3.4 Neurology-specific investigations and treatment

During admission, all NID patients with central nervous system (CNS) syndromes (109/118; 92 %) had dedicated brain or spine neuroimaging (CT and/or MRI) performed, as appropriate. The NID patients who presented with neuromuscular disorders underwent nerve conduction studies and electromyography as part of their diagnostic assessment. All NID patients received appropriate treatment for their infectious disease during admission.

### 3.5 Outcome

The duration of admission for NID patients was significantly longer than for non-NID patients (median: 16 days, [IQR 9–28] versus 10 days, [6–18]; *p* < 0.001). Six out of the 118 NID patients (5 %) demised in hospital, which was similar to the 4 % (*n* = 24) mortality in the non-NID group. At discharge, only 2 of the 112 surviving NID patients had a modified Rankin Scale (mRS) score of 0, indicative of full recovery. Sixty-three percent (*n* = 71) required further inpatient care at a primary or secondary level medical facility. Two patients died within three months after discharge. Of 41 patients with an NID who were followed up at GSH three months after discharge, 39 still had residual symptoms or disability (mRS ≥1) related to their neurological infection.

## 4 Discussion

This study assessed the burden of NIDs on the specialist inpatient neurology service at GSH, a 975-bed tertiary and quaternary level hospital, which includes 20 dedicated neurology beds.

Over a two-year period, NID patients comprised 15 % of the total patient population admitted to the neurology service. This is a considerably higher NID burden than noted in Brazil [11] and in several tertiary centres in developed settings such as the United States and Europe where the reported proportions of NID range between 2 and 9 % [8,12,13]. Studies from Africa report variable findings: Research from Cameroon describes a frequency of CNS infections ranging from 2 % to 3 % during their 5-year study period [14], a study from Nigeria showed a proportion of NID cases of 22 % [15], while in a study from Tanzania 33 % of medical patients had a neurological infection [16]. Our patients were younger (median age 38-years) than what has been described in the United States (mean age 43 years [8]) and Europe (median age 51 years [12]), but similar in age to those of Nigeria (mean age 34 years [15]). The higher prevalence of NID and the younger age generally observed in developing countries is not surprising and reflect the higher infectious diseases burden in these settings, compounded by HIV associated immunosuppression [17].

Most patients diagnosed with a NID required prolonged admission, with 56 % of these patients admitted to the neurology ward for more than two weeks. Our median admission duration for NID patients was significantly longer than that in non-NID patients (median: 16 vs. 10 days). Although the 5 % inpatient mortality rate was relatively low, equal to that noted in Germany [12] and comparable to the 9 % case fatality rate from a multinational study in Europe and Asia [13], morbidity was high. Only 2 % of NID patients had made a full recovery (mRS = 0), and 63 % required further stepdown care upon discharge from hospital. Taken together, these findings emphasise the strain that NID places on our healthcare system.

HIV, TB, and other opportunistic infections associated with HIV accounted for 74 (56 %) of the 132 NID cases in this study. Among all HIV-positive NID patients in this study, 61 % (*n* = 40) were either not receiving ART or had a detectable HIV viral load suggestive of ineffective ART. Other studies from Africa report even lower prescription of ART in patients with NID; in a study from Cameroon only 38 % of HIV-positive NID patients had been on regular ART prior to hospital admission [14].

We considered whether the Covid-19 pandemic may have contributed to the low number of patients on ART. All patients admitted with a known diagnosis of HIV, and who had been diagnosed with HIV during the COVID-19 pandemic, had been appropriately started on ART prior to admission. Among the 121 HIV-positive patients admitted during the study period, 16 had interrupted ART treatment. A quarter (n = 4) of these treatment interruptions had occurred during the COVID-19 lockdown period. There is an extensive ART rollout programme in South Africa, but as our findings show, untreated HIV is still common, consistent ART provision is still not optimal and HIV case finding has to remain an important healthcare priority.

South Africa is a TB endemic country, with one of the highest incidence rates of TB worldwide; estimated at over 500 cases per 100,000 people per year [18]. Thus, the burden of neurological TB in our patient population is not unexpected. TB was the most frequently encountered NID aetiology in this study, diagnosed in over a quarter (27 %) of cases. TB affects both HIV-positive and HIV-negative patients. However, in our patient population, 31 % of patients with neurological TB were immunocompetent, without HIV or another immunosuppressive condition. This contrasts with data from countries such as Brazil, where neurological TB was only observed in immunosuppressed patients [11]. The burden of neurological manifestations due to *M. tuberculosis* is likely underestimated: In our hospital, patients with bony TB spine (Pott's disease) are managed by the orthopaedic spinal units and not in the neurology ward. Hence the fact that only two patients with Pott's disease were identified in our patient sample probably reflects this referral bias. Furthermore, although TB meningitis was diagnosed in 28 % of patients with neurological TB, only a minority of TB meningitis cases in South Africa are referred for tertiary-level neurology care; these patients are usually managed in general medicine wards or in secondary level hospitals. In a study from Cape Town, performed at a district-level facility, most meningitis cases (120/211) were due to tuberculous meningitis [19]. The diagnosis of CNS TB is known to be difficult [20], and in our study a definite TB diagnosis was only made in 3/36 patients.

Neurosyphilis was responsible for a fifth of NID cases in this study. This is a far higher proportion than in other global tertiary medical centres: In North and South American studies, a prevalence of neurosyphilis of 3 % to 4.5 % was observed in their neuroinfectious patients [8,11]. In a German study, only one case of neurosyphilis was diagnosed among 376 neuroinfectious patients [12], no neurosyphilis patients were identified in a study from Cameroon [14] and in centres from Asia and Europe, neurosyphilis contributed less than 0.001 % of CNS infections [13]. The minority of patients with neurosyphilis in our study were HIV-coinfected (33 %); a percentage similar to the 35 % HIV co-infection rate previously described in patients with neurosyphilis at our hospital facility [21]. Neurosyphilis was noticeably more common in males than in females, with 74 % of neurosyphilis cases diagnosed in males. This mirrors findings from the Netherlands, where 75 % of neurosyphilitic cases were men. In South Africa, pregnant women are screened for syphilis with a RPR test as part of the national antenatal programme [22]. However, there is no separate national broad screening programme for men. Thus, women of childbearing age are more likely to be screened for syphilis and consequently more likely to receive timely treatment for early syphilis than their male peers. This relative lack of early opportunistic syphilis testing, diagnosis, and early treatment in men may be factors contributing to significantly higher rates of neurosyphilis in males.

SARS-CoV-2 related neurological conditions were diagnosed in 17 % of NID cases. Only one definitive diagnosis was made. In the remaining patients, other causes were excluded after extensive investigation. A single patient had been vaccinated against COVID-19 prior to admission. Among the remaining 22 patients, 13 had presented prior to COVID-19 vaccination becoming available to them. All manifestations that we report have been previously described in COVID-19 cases, including encephalitis, stroke, seizures, and Guillain-Barre syndrome [23,24]. However, the causal contribution of SARS-CoV-2 to neurological presentations is still uncertain [25] and therefore the diagnosis may have been inaccurate in some of our patients.

Of the 132 neurological infections diagnosed in our study, only 17 % could be classified as having a definite diagnosis. Comparatively, the microbiological confirmation of infectious pathogens in a large multinational study of centres in Europe and North America was 42 % [13], probably reflecting different causative organisms.

Bacterial meningitis was the most common NID in the aforementioned multinational study [13]. Similar findings were noted at other tertiary centres in developed countries [8,12,26], with bacterial meningitis, viral meningitis and meningoencephalitis constituting the majority of their NID cases. In contrast, very few patients with viral and bacterial meningitis were admitted to our neurology service. As bacterial meningitis is often readily diagnosed on CSF testing, this likely contributes to the higher rate of microbiologically confirmed NID cases in such centres. The minimal number of meningitis cases in our study also highlights the scarcity of specialist neurological and neuroinfectious disease centres in many low- and middle-income countries. High-income countries average 0.73 neurology beds per 10,000 people, compared to the 0.03 neurology beds per 10,000 population of low-income countries [27]. In Africa, there is estimated to be only one neurologist per 3 million population, compared to Europe with a neurologist per 20,000 people [27].

Restricted access to histopathological services during the COVID-19 pandemic may also have contributed to the reduced number of patients with a confirmed microbiological diagnosis. Throughout much of the pandemic, surgical services at our institution were limited to emergencies only. As a result, patients with a presumptive diagnosis of a NID were generally managed empirically based on clinical and radiological features, without confirmatory histopathological evaluation. Academic post-mortem procedures were also severely restricted during this period. Only 2 % of our patients underwent CNS biopsy, and no autopsies were performed. By comparison, data from the Johns Hopkins Medical Institutions indicate that 6 % of their NID patients underwent either CNS biopsy or post-mortem autopsy to support diagnostic confirmation [8].

A specific strength of this research is that ∼90 % of all patients admitted to the neurology ward were tested for syphilis and HIV; including all patients in the NID group. Most of the available data on the prevalence of syphilis in South Africa has been obtained from pregnant women who attended antenatal clinics. Based on the most recent national data, the prevalence of syphilis among pregnant women in South Africa is 2.1 % [22]. When excluding the 27 patients diagnosed with neurosyphilis, the proportion of patients with active syphilis in our overall study population was 2.7 %. In South Africa, the HIV burden is disproportionately higher in females than in males [28]. This was also reflected in our study population, where, in both the NID and non-NID disease groups, females had proportionally higher HIV infection rates than males. The proportion of HIV positive patients (17 %), among the 730 patients who were tested for HIV, is comparable to the national South African HIV prevalence estimate of 14 % [28].

The main limitation of this study is its retrospective nature with all the biases associated with such a study. However, detailed clinical documents and investigation results were available for all patients. The lack of laboratory confirmation for some infections affected the diagnostic certainty in patients without microbiological confirmation of infection. For example, as only three patients had microbiologically confirmed TB, a TB diagnosis was made on the basis of possibility and probability in the majority of cases. Another limitation is intrinsic referral and inclusion bias in our setting, as most patients with an NID who do not require special investigations (such as those with meningitis) are managed in secondary level hospitals.

## 5 Conclusion

This study provides important insights into the burden and characteristics of NIDs in a tertiary hospital inpatient neurology service in South Africa. The findings highlight the importance of HIV and syphilis testing, the need for improved ART treatment strategies, and increased investment in neurological services to reduce the burden of neuroinfectious diseases and improve patient outcomes. Further research is needed to better understand the underlying factors contributing to the high burden of neuroinfectious diseases in this population and to develop targeted early interventions to address these challenges.

## Research support

This work is based on the research supported in part by the National Research Foundation of South Africa (Grant Number 132051).

## Relationships

There are no additional relationships to disclose.

## Other activities

There are no additional activities to disclose.

## CRediT authorship contribution statement

**Katryn Oosthuizen:** Writing – original draft, Investigation, Data curation. **Suzaan Marais:** Supervision, Conceptualization, Writing – review & editing.

## Ethics approval

Research for this study was approved by the Human Research Ethics Committee of the.

Faculty of Health Sciences at the University of Cape Town (HREC REF: 404/2022) and by.

Groote Schuur Hospital.

Consent of publication

The manuscript has not been published elsewhere. All authors have read and approved the submitted manuscript.

## Declaration of competing interest

None.

## Figures and Tables

**Table A1:** Diagnostic score for classification of tuberculous meningitis (5)

Clinical criteria (Maximum category score=6)	Diagnostic score
Symptom duration of more than 5 days	4
Systemic symptoms suggestive of tuberculosis (one or more of the following): weight loss, night sweats, or persistent cough for more than 2 weeks	2
History of recent (within past year) close contact with an individual with pulmonary tuberculosis	2
Focal neurological deficit (excluding cranial nerve palsies)	1
Cranial nerve palsy	1
Altered consciousness	1
CSF criteria (Maximum category score=4)	
Clear appearance	1
Cells: 10–500 per μL	1
Lymphocytic predominance (>50%)	1
Protein concentration greater than 1 g/L	1
CSF to plasma glucose ratio of less than 50% or an absolute CSF glucose concentration less than 2·2mmol/L	1
Cerebral imaging criteria (Maximum category score=6**)**	
Hydrocephalus	1
Basal meningeal enhancement	2
Tuberculoma	2
Infarct	1
Pre-contrast basal hyperdensity	2
Evidence of tuberculosis elsewhere (Maximum category score=4**)**	
Chest radiograph suggestive of active tuberculosis: Signs of tuberculosis=2; miliary tuberculosis=4	2/4
CT/ MRI/ ultrasound evidence for tuberculosis outside the central nervous system (CNS)	2
AFB identified or *Mycobacterium tuberculosis* cultured from another source (sputum, lymph node, gastric washing, urine, blood culture)	4
Positive commercial *M tuberculosis* nucleic acid amplification test from extra-neural specimen	4

**Table A1:** Preferred diagnostic viral encephalitis test according to suspected aetiology (14,15)

Viral pathogen	Diagnostic test
Herpes simplex virus (HSV-1/HSV-2)	CSF PCR
Varicella zoster virus (VZV)	CSF specific IgG
Enterovirus	Stool, throat or CSF PCR
Epstein-Barr virus (EBV)	Serum EBV capsid antigen IgG and IgM (VCA) and EBV nuclear antigen IgG (EBNA)
Measles	CSF specific IgG
Cytomegalovirus (CMV)	CSF PCR or CSF-specific IgM
Influenza	Culture, antigen test, PCR of respiratory secretions

## Appendix A Diagnostic criteria used to classify neuroinfectious diseases

**1. Neurosyphilis diagnostic criteria**

Laboratory tests for syphilis include: 1) Nontreponemal (venereal disease research laboratory [VDRL] and rapid plasma reagin [RPR]) tests, and 2) Treponemal (fluorescent treponemal antibody absorption [FTA-abs]and *Treponema Pallidum* antibody [TpAb]) tests (1,2).

The VDRL test is a highly specific non-treponemal antibody test. In the appropriate clinical context, a positive CSF VDRL result (in a specimen not contaminated with blood) confirms the diagnosis of neurosyphilis. However, the sensitivity of the VDRL test is suboptimal (30-70%). Conversely, the CSF FTA-abs test is very sensitive (>90%), but with a lower specificity. Since antibodies can persist lifelong after adequate treatment or intrinsic immune clearance of syphilis, a positive FTA-abs test cannot confirm current active infection (1, 2).

The diagnosis of neurosyphilis is thus based on the evaluation of a patient’s clinical features, cerebrospinal fluid (CSF) parameters and on the presence of intrathecal treponemal and/or non-treponemal antibodies.

Neurosyphilis diagnostic certainty was classified as follows (3,4):

Definite neurosyphilis

A compatible neurological manifestation consistent with neurosyphilis AND a reactive cerebrospinal fluid (CSF) VDRL (in the context of an atraumatic lumbar puncture)

Probable neurosyphilis

A compatible neurological manifestation consistent with neurosyphilis; and criterion 1 and 2 below are met AND either criterion 3 or 4 below is met:

1) Reactive TPAB and RPR serology
2) Reactive CSF FTA-abs test
3) Raised CSF leukocytes (>5 cells/μL in human immunodeficiency virus (HIV)-uninfected and >20 cells/μL in HIV-infected persons)
4) CSF protein >0.45 g/l

Possible neurosyphilis

A compatible neurological manifestation consistent with neurosyphilis AND criterion 1 and 2 below are met AND either criterion 3 or 4 below is met

1) Reactive TPAB serology
2) A reactive serum RPR or CSF FTA-abs test (if both tests were not performed)
3) Raised CSF leukocytes (>5 cells/μL in HIV-uninfected, >20 cells/μL in HIV-infected persons)
4) CSF protein >0.45 g/L

**2a. Tuberculous meningitis diagnostic criteria (5)**

Definite tuberculous meningitis

Fulfilment of either criterion 1 OR 2 below:

1) Suggestive symptoms or signs of meningitis, plus one or more of the following:
  a) Acid-fast bacilli detected in the CSF
  b) *Mycobacterium tuberculosis* cultured from the CSF
  c) CSF positive commercial nucleic acid amplification test
2) Acid-fast bacilli identified on a tissue sample, with histological features consistent with tuberculosis of the brain or spinal cord, in the context of symptoms or signs of meningitis and suggestive CSF changes, or visible meningitis (on autopsy).

Probable tuberculous meningitis

Symptoms and signs suggestive of meningitis plus:

1) A total diagnostic score (refer to Table A1) of 10 or more points (when brain imaging is not available) or 12 or more points (when brain imaging is available)
2) Exclusion of alternative diagnoses

At least 2 points obtained from CSF or cerebral imaging criteria

Possible tuberculous meningitis

Symptoms and signs suggestive of meningitis plus:

1) A total diagnostic score (refer to Table A1) of 6–9 points (when brain imaging is not available) or 6–11 points (when brain imaging is available)
2) Exclusion of alternative diagnoses

**2b. Spinal tuberculosis diagnostic criteria (6)**

Definite spinal tuberculosis

A compatible neurological manifestation and positive tuberculosis (TB) results from microscopy, culture, histology, or Xpert MTB/RIF test of any central nervous system (CNS) sample.

Probable spinal tuberculosis

Compatible neurological and MRI radiological features, along with one of the following:

1) Concurrent TB meningitis
2) Spondylitis with the presence of tuberculosis outside the CNS
3) Myeloradiculitis with features of extra-CNS TB, along with exclusion of other causes by CSF analysis

Possible spinal tuberculosis

Compatible neurological and MRI radiological features, without microbiological evidence of TB, and clinical or radiological improvement in neurological symptoms/signs following the initiation of TB treatment

**2c. Intracranial tuberculoma diagnostic criteria (7,8)**

Definite intracranial tuberculoma

A compatible intracranial lesion on computed tomography (CT) or magnetic resonance imaging (MRI), and positive TB results from either microscopy, culture, histology, or Xpert MTB/RIF testing on biopsy sample of the lesion.

Probable intracranial tuberculoma

A compatible intracranial lesion on neuroimaging (CT or MRI), and one of the following:

1) Concurrent TB meningitis
2) Concurrent spinal tuberculosis
3) Features of extra-CNS TB, and clinical or radiological improvement in neurological symptoms/signs after commencing TB treatment

Possible intracranial tuberculoma

A compatible intracranial lesion on neuroimaging (CT or MRI), and clinical or radiological improvement in neurological symptoms/signs after commencing TB treatment

**3. Neurocysticercosis diagnostic criteria (8,9)**

Definite neurocysticercosis

1) One absolute criterion.
2) Two major neuroimaging criteria plus any clinical criteria. One major and one confirmative neuroimaging criteria plus any clinical criteria.
3) One major neuroimaging criterion plus two clinical criteria (including at least one major clinical criterion), together with the exclusion of other pathologies producing similar radiological features.

Probable neurocysticercosis

1) One major neuroimaging criterion plus any two clinical criteria
2) One minor neuroimaging criterion plus at least one major clinical criterion

Absolute criteria

1) Histological demonstration of the parasite from biopsy of a lesion in the brain or spinal cord
2) Visualisation of subretinal cysticercus
3) Conclusive demonstration of a scolex within a cystic lesion on neuroimaging

Neuroimaging criteria

Major neuroimaging criteria:

1) Cystic lesions without a discernible scolex
2) Enhancing lesions
3) Multilobulated cystic lesions in the subarachnoid space
4) Typical parenchymal brain calcifications

Confirmative neuroimaging criteria

1) Resolution of cystic lesions after treatment for neurocysticercosis
2) Spontaneous resolution of single small enhancing lesions (this criterion is invalid if any corticosteroids were administered)
3) Migration of ventricular cysts evident on sequential neuroimaging studies

Minor neuroimaging criteria:

1) Obstructive hydrocephalus or abnormal enhancement of the basal leptomeninges

Clinical/exposure criteria

Major clinical/exposure criteria:

1) Detection of specific anticysticercal antibodies or detection of cysticercal antigens
2) Cysticercosis outside the central nervous system
3) Evidence of a household contact with *T. solium* infection

Minor clinical/exposure criteria:

1) Clinical manifestations suggestive of neurocysticercosis (seizures, chronic headaches, focal neurologic deficits, raised intracranial pressure and cognitive decline).
2) Individuals coming from or living in an area where cysticercosis is endemic.

**4. Progressive multifocal leukoencephalopathy (PML) diagnostic criteria (10)**

Definite PML

Criterion 1-3 below are met

1) Compatible clinical features
2) Compatible neuroimaging findings: Hypodense white matter lesions on CT brain; high signal intensity grey-white junction or brainstem white matter lesions on MRI T2/FLAIR images; minimal mass effect, +/- enhancement
3) A positive CSF polymerase chain reaction (PCR) for JC (John Cunningham) virus

Probable PML

1) Criterion 1 and 3 below are met OR criterion 2 and 3 below are met
2) Compatible clinical features
3) Compatible imaging findings CSF PCR positive for JC virus

Possible PML

Criterion 1 and 2 below are met OR only criterion 3 below is met

1) Compatible clinical features
2) Compatible imaging findings
3) CSF PCR positive for JC virus

**5. HIV-associated neurocognitive disorder (HAND) diagnostic criteria**

The most frequently utilised criteria for classifying cognitive impairment in persons living HIV with are the HIV–associated neurocognitive disorders (HAND) criteria (11). Though these criteria were intended for use in the research setting, the terminology is also frequently applied when describing the clinical burden of disease. However, it has been argued that HAND research criteria should not be extrapolated to evaluating disease in a clinical context, as when applying the HAND criteria, up to 60% of persons living with HIV meet criteria for neurocognitive impairment (12). These figures do not equate with clinical observations, where cognitive disorders have a relatively low frequency in persons living with HIV (12). An alternative approach to characterise cognitive impairment in persons living with HIV has been suggested, placing a more significant emphasis on the multifactorial nature of cognitive test performance (12). However, criteria for diverse global populations that are applicable to both research and clinical settings still need to be developed

HIV-1-associated mild neurocognitive disorder (MND)

1) Acquired impairment in cognitive functioning involving at least two of the following domains: Language; attention/working memory; abstraction/executive function; memory (learning; recall); speed of information processing; sensory-perceptual ability, motor skills
2) The cognitive impairment produces some interference in daily functioning at least one of the following):
  a. Self-report of reduced mental acuity, or impaired work or social functioning.
  b. Observation by knowledgeable others that the individual has undergone at least mild decline in mental acuity with resultant impairment in work, homemaking, or social functioning.
3) The cognitive impairment does not meet criteria for delirium or dementia.
4) There is no evidence of another pre-existing cause for neurocognitive impairment (such as major depression or substance dependence)

HIV-associated dementia (HAD)

1) Acquired impairment in cognitive functioning
2) The cognitive impairment produces marked interference with day-to-day functioning (work, home life, social activities)
3) The pattern of cognitive impairment does not meet criteria for delirium
4) There is no evidence of another, pre-existing cause for the dementia

**6. Cerebral toxoplasmosis diagnostic criteria (13)**

Definite cerebral toxoplasmosis

Fulfilment of criterion 1, 2 and either criterion 3 or 4 below:

1) Compatible clinical syndrome (clinical manifestations include headache, focal neurological deficit, altered mentation, seizures, psychomotor changes, cranial nerve palsy, ataxia, visual abnormalities, intracranial hypertension syndrome and involuntary movements)
2) Typical brain lesions on radiological imaging
3) Evidence of Toxoplasma gondii deoxyribonucleic acid (DNA) in CSF by nucleic acid amplification assays.
4) Brain biopsy, or post-mortem examination, showing evidence of T gondii on immunoperoxidase staining or on haematoxylin and eosin stains for T gondii tachyzoites

Probable cerebral toxoplasmosis

Fulfilment of criterion 1, 2 and 3 below:

1) A compatible clinical syndrome
2) Typical brain lesions on radiological imaging
3) Unequivocal radiological or clinical response to 14 days of only anti-toxoplasma therapy

Possible cerebral toxoplasmosis

Fulfilment of criterion 1, 2 and 3 below:

1) A compatible clinical syndrome
2) Typical brain lesions on radiological imaging
3) Presence of serum T gondii IgG antibodies

**7a. Viral encephalitis diagnostic criteria (14,15)**

Definite viral encephalitis

Fulfilment of criterion 1, and either criterion 2 or 3 below:

1) Altered mental status (defined as decreased or altered level of consciousness, lethargy, personality change) lasting ≥24 hours with no alternative cause identified
2) Pathologic confirmation of brain inflammation consistent with encephalitis
3) Pathologic, microbiologic, or serologic evidence of infection with an organism strongly associated with encephalitis from an appropriate clinical specimen (see table A2 below)

Probable viral encephalitis

Altered mental status lasting ≥24 hours with no alternative cause identified AND three or more of the below listed minor criteria

Possible viral encephalitis

Altered mental status lasting ≥24 hours with no alternative cause identified AND two of the below listed minor criteria

Minor criteria

1) Documented fever ≥38 °C
2) Seizures not fully attributable to a pre-existing seizure disorder
3) New onset of focal neurologic findings
4) CSF leukocyte count ≥5 cells/μL
5) Abnormality of brain parenchyma on neuroimaging suggestive of encephalitis
6) Abnormality on electroencephalography (EEG) consistent with encephalitis and not attributable to another cause; EEG abnormalities reported in cases of encephalitis range from nonspecific generalised slowing to distinctive patterns suggestive of specific entities

**7b. Cytomegalovirus (CMV) diagnostic criteria (16)**

Definite CMV neuroinfection

Compatible neurologic manifestations of CMV infection (including encephalitis, ventriculitis, myelitis, retinitis, radiculoganglionitis, and peripheral neuropathies) and detection of CMV in CNS tissue

Probable CMV neuroinfection

CNS symptoms plus detection of CMV in CSF without visible contamination of blood (“bloody tap”), plus abnormal imaging results or evidence of encephalitis on EEG

**8. Bacterial meningitis diagnostic criteria (17,18)**

Definite bacterial meningitis:

Fulfilment of any of the following criteria:

1) A CSF Gram-stained smear positive for a bacterial pathogen^a^
2) A positive CSF culture for a bacterial pathogen^a^
3) A positive CSF polymerase chain reaction assay for *Neisseria meningitidis*^b^
4) CSF pleocytosis (>7 white blood cells/μL) and identification on blood of *Neisseria meningitidis*, *Streptococcus pneumoniae* or *Haemophilus influenzae* type b
5) Identification on CSF^b^ of *S. pneumoniae, H. influenzae,* Group B *Streptococcus, Listeria monocytogenes, Staphylococcus aureus*^a^ *or Escherichia coli* ^a^

Probable bacterial meningitis:

Fulfilment of all the following criteria:

1) Sudden onset of fever (>38.0 °C axillary)
2) Neck stiffness or another meningeal sign, including meningeal enhancement (if neuroradiological imaging i.e., CT or MRI was performed)
3) CSF leukocyte count >10 cells/μL
4) Clinical response to antibiotic treatment.

Possible bacterial meningitis

Fulfilment of all the following criteria:

1) Sudden onset of fever (>38.0 °C axillary)
2) Neck stiffness or another meningeal sign, including neuroradiological imaging demonstrating meningeal enhancement
3) Contra-indication or inability to obtain cerebrospinal fluid for analysis
4) Clinical response to antibiotic treatment.
5) ^a^ Bacterial pathogens commonly associated with contamination (such coagulase-negative staphylococci, bacilli, and micrococci) will only be deemed to be the causative agent for meningitis if a repeat CSF sample or a sterile blood culture yields the same organism. ^b^ The bacterial meningitis screen performed at Groote Schuur Hospital encompasses: PCR testing for *Streptococcus pneumoniae, Haemophilus influenzae* and *Neisseria meningitidis*

**9. Diagnostic criteria for severe acute respiratory syndrome coronavirus 2 (SARS-CoV-2) associated neurological syndromes (19)**

Definite SARS-CoV-2 neurological syndrome

Fulfilment of all the following criteria:

1) Compatible neurological syndrome^a-g^ in temporal relationship with SARS-CoV-2 infection
2) SARS-CoV-2 virus detected in cerebrospinal fluid or brain tissue
3) Absence of an alternative aetiology and/or risk factors

Probable SARS-CoV-2 neurological syndrome

Fulfilment of both criterion 1 and 4, and fulfilment of either criterion 2 or 3 below:

1) Compatible neurological syndrome^a^ in temporal relationship with SARS-CoV-2 infection and
2) SARS-CoV-2 virus detected in respiratory or other non-CNS sample
3) Evidence of SARS-CoV-2-specific serum antibody, compatible with acute infection
4) Absence of an alternative aetiology and/or risk factors

Possible SARS-CoV-2 neurological syndrome

Fulfilment of both criterion 1 and 4, and fulfilment of either criterion 2 or 3 below:

1) Compatible neurological syndrome^a^ in temporal relationship with SARS-CoV-2 infection and
2) SARS-CoV-2 virus detected in respiratory or other non-CNS sample
3) Evidence of SARS-CoV-2-specific serum antibody, compatible with acute infection
4) Presence of alternative aetiology and/or risk factors

^a^**Encephalitis:** New-onset encephalopathy with evidence of CNS inflammation (CSF white cell count >4 cells/ml, protein >0.45g/dL, or MRI/CT consistent with parenchymal inflammation) and/or clinical or electrophysiological evidence of parenchymal brain involvement (such as focal neurological deficit, new-onset seizure, or focal changes on EEG).

^b^ Meningitis: One or more of headache, fever, meningism, with evidence of CNS inflammation (such as CSF white cell count >4 cells/ml, protein >0.45g/dL, or MRI/CT consistent with meningeal inflammation). Meningitis with evidence of brain parenchymal involvement indicates meningoencephalitis.

^c^ Myelitis: Weakness and/or sensory disturbance of the limbs, clinically attributable to a spinal cord lesion, with evidence of CNS inflammation (CSF white cell count >4/ml, protein >0.45g/dL, oligoclonal bands, or MRI consistent with spinal inflammation.

^d^ Guillain-Barre syndrome (GBS) and GBS variants: Clinical, electrophysiological and laboratory findings attributable to an acute immune-mediated demyelinating peripheral nerve pathology.

^e^ Neuropathy not attributable to another neurological disease.

^f^ Myopathy and myositis: Motor symptoms and/or signs attributable to muscle damage (myopathy) and/or inflammation (myositis) with elevation of peripheral muscle enzymes (e.g., creatinine kinase-CK).

^g^ Cerebrovascular disease and stroke: Symptoms, signs, and neuroimaging findings consistent with an occlusion or insult affecting the vasculature of the central nervus system. This includes:

• Transient ischaemic attack (symptoms/signs <24 hours)
• Ischaemic stroke
• Intracerebral or subarachnoid haemorrhage, not attributable to any other cause
• Cerebral vasculitis
• Cerebral venous sinus thrombosis

**10. Neuroschistosomiasis diagnostic criteria (20,21)**

Definite neuroschistosomiasis

A compatible neurological manifestation consistent with neuroschistosomias and demonstration of schistosomal ova or worms in the CNS

Probable neuroschistosomiasis

A compatible neurological manifestation consistent with neuroschistosomiasis and criterion 1 and 2 below are met:

1) Demonstration of schistosomal infection by parasitological methods, either by the identification of schistosomal ova on stool /rectal biopsy or by the demonstration of anti-schistosomal serum antibodies
2) Spinal MRI imaging demonstrating features of a low thoracic or lumbar/sacral inflammatory myelopathy

Possible neuroschistosomiasis

A compatible neurological manifestation consistent with neuroschistosomiasis and criterion 1 to 3 below are met:

1) Spinal MRI imaging demonstrating features of a low thoracic or lumbar/sacral inflammatory myelopathy
2) A history of exposure to open freshwater in a schistosomal endemic region
3) Exclusion of alternative diagnoses

**11. Diagnostic criteria for other neuroinfectious diseases:**

Diagnostic criteria for other neuroinfectious diseases were based on published international guidelines. Where no clear diagnostic consensus criteria were available, the following criteria from research conducted by the John Hopkins Medical Institution was utilised (22).

Definite neuroinfection:

1) A pathogen was identified on tissue biopsy or cerebrospinal fluid (CSF) microscopy, culture, antibody detection or PCR.
2) In the appropriate clinical setting, a compatible neuroinfectious pathogen was identified on blood, urine or sputum by culture, light microscopy, antigen testing or PCR

Probable neuroinfection:

1) A patient with a pathognomonic infectious clinical syndrome, such as Varicella zoster associated Ramsey-Hunt syndrome, once other aetiological causes for the clinical syndrome are excluded.
2) A patient with radiological findings on neuroimaging consistent with a clinical syndrome of a neuroinfectious pathogen
3) A patient with at least one feature of CNS infection (fever, altered mentation, focal neurologic deficits, fever), CSF pleocytosis and a positive response to appropriate antimicrobial therapy

## Appendix B References for Appendix A

1. Davis LE, Schmitt JW. Clinical significance of cerebrospinal fluid tests for neurosyphilis. Ann Neurol. 1989;25(1):50–5. doi: 10.1002/ana.410250108

2. Henao-Martínez AF, Johnson SC. Diagnostic tests for syphilis: New tests and new algorithms. Neurol Clin Pract. 2014;4(2):114-122. doi: 10.1212/01.CPJ.0000435752.17621.48

3. Dumaresq J, Langevin S, Gagnon S, Serhir B, Deligne B, Tremblay C, et al. Clinical prediction and diagnosis of neurosyphilis in HIV-infected patients with early syphilis. J Clin Microbiol. 2013;51(12):4060-6. doi: 10.1128/JCM.01989-13

4. Workowski KA, Bolan GA. Centers for disease control and prevention. Sexually transmitted diseases treatment guidelines: Syphilis. 2015. Available from: https://www.cdc.gov/std/tg2015/syphilis.htm#Neurosyphilis [Accessed 17 July 2023]

5. Marais S, Thwaites G, Schoeman JF, Török E, Misra UK, Prasad K, et al. Tuberculous meningitis: a uniform case definition for use in clinical research. Lancet Infect Dis. 2010;10(11):803-12. doi: 10.1016/S1473-3099(10)70138-9

6. Marais S, Roos I, Mitha A, Mabusha SJ, Patel V, Bhigjee AI. Spinal tuberculosis: Clinicoradiological findings in 274 Patients. Clin Infect Dis. 2018;67(1):89-98. doi: 10.1093/cid/ciy020

7. Marais S, Van Toorn R, Chow FC, Manesh A, Siddiqi OK, Figaji A, et al. Management of intracranial tuberculous mass lesions: how long should we treat for? Wellcome Open Res. 2019;4:158. doi: 10.12688/wellcomeopenres.15501.2

8. Diker, S., Ruso, D.Ö., Bayraktar, N. et al. Intracranial tuberculomas or neurocysticercosis: differentiated by cervical lymph node pathology. Egypt J Neurol Psychiatry Neurosurg. 2022;58:117. doi: 10.1186/s41983-022-00554-x

9. Del Brutto OH, Nash TE, White AC Jr, Rajshekhar V, Wilkins PP, Singh G, et al. Revised diagnostic criteria for neurocysticercosis. J Neurol Sci. 2017;372:202-210. doi: 10.1016/j.jns.2016.11.045

10. Berger JR, Aksamit AJ, Clifford DB, Davis L, Koralnik IJ, Sejvar JJ, et al. PML diagnostic criteria: Consensus statement from the AAN Neuroinfectious Disease Section. Neurology. 2013;80(15):1430-8. doi: 10.1212/WNL.0b013e31828c2fa1

11. Antinori A, Arendt G, Becker JT, Brew BJ, Byrd DA, Cherner M, et al. Updated research nosology for HIV-associated neurocognitive disorders. Neurology. 2007;69(18):1789-99. doi: 10.1212/01.WNL.0000287431.88658.8b

12. Nightingale S, Dreyer AJ, Saylor D, Gisslén M, Winston A, Joska JA. Moving on from HAND: Why we need new criteria for cognitive impairment in persons living with human immunodeficiency virus and a proposed way forward. Clin Infect Dis. 2021;73(6):1113-1118. doi: 10.1093/cid/ciab366

13. Vidal JE. HIV-related cerebral toxoplasmosis revisited: Current concepts and controversies of an old disease. J Int Assoc Provid AIDS Care. 2019 Jan-Dec;18:2325958219867315. doi: 10.1177/2325958219867315

14. Venkatesan A, Tunkel AR, Bloch KC, Lauring AS, Sejvar J, Bitnun A, et al. International encephalitis consortium. Case definitions, diagnostic algorithms, and priorities in encephalitis: consensus statement of the international encephalitis consortium. Clin Infect Dis. 2013;57(8):1114-28. doi: 10.1093/cid/cit458

15. Costa BK da, Sato DK. Viral encephalitis: a practical review on diagnostic approach and treatment. Jornal de Pediatria. 2020;96:12–9. doi: 10.1016/j.jped.2019.07.006

16. Ljungman P, Boeckh M, Hirsch HH, Josephson F, Lundgren J, Nichols G, et al. Definitions of cytomegalovirus infection and disease in transplant patients for use in clinical trials. Clin Infect Dis. 2017;64(1):87-91. doi: 10.1093/cid/ciw668

17. World Health Organization. Managing meningitis epidemics in Africa: a quick reference guide for health authorities and health-care workers, Revised 2015 [Internet]. Document number: WHO/HSE/GAR/ERI/2010.4 Rev.1. Geneva: World Health Organization; 2015. p. 17. Available from: https://apps.who.int/iris/handle/10665/154595 [Accessed 22 October 2023]

18. Brivet FG, Ducuing S, Jacobs F, Chary I, Pompier R, Prat D, et al. Accuracy of clinical presentation for differentiating bacterial from viral meningitis in adults: A multivariate approach. Intensive Care Med. 2005;31(12):1654–60. doi: 10.1007/s00134-005-2811-1

19. Ellul MA, Benjamin L, Singh B, Lant S, Michael BD, Easton A, et al. Neurological associations of COVID-19. Lancet Neurol. 2020;19(9):767-783. doi: 10.1016/S1474-4422(20)30221-0

20. Nascimento-Carvalho CM, Moreno-Carvalho OA. Neuroschistosomiasis due to Schistosoma mansoni: A review of pathogenesis, clinical syndromes and diagnostic approaches. Rev Inst Med Trop Sao Paulo. 2005;47(4):179-84. doi: 10.1590/s0036-46652005000400001

21. Adeel AA. Spinal cord schistosomiasis. Sudan J Paediatr. 2015;15(2):23-8

22. Tan K, Patel S, Gandhi N, Chow F, Rumbaugh J, Nath A. Burden of neuroinfectious diseases on the neurology service in a tertiary care center. Neurology. 2008;71(15):1160-6. doi: 10.1212/01.wnl.0000327526.71683.b7
